# Postoperative Aspergillus Endophthalmitis With Iris Granuloma: A Case Report

**DOI:** 10.7759/cureus.44945

**Published:** 2023-09-09

**Authors:** Aysha Aloqab, Valmore A Semidey

**Affiliations:** 1 Vitreoretinal Division, King Khaled Eye Specialist Hospital, Riyadh, SAU; 2 Ophthalmology, Bahrain Defence Force Hospital, Bahrain, BHR

**Keywords:** iris granuloma, aspergillus, aspergillus endophthalmitis, vitrectomy, phacoemulsification, iris, granuloma, fungal, endophthalmitis

## Abstract

This report describes a rare case of a fungal iris granulomatous lesion in a 67-year-old female who underwent cataract surgery in the left eye and, one month later, developed culture-positive postoperative *Aspergillus *endophthalmitis. On initial presentation in the emergency room, slit-lamp examination of the left eye revealed subconjunctival hemorrhage, 360-degree subconjunctival hemorrhage, mild corneal edema with Descemet’s folds, a deep anterior chamber with a fibrinous reaction causing pupillary membrane formation, and an in situ intraocular lens with no view of the fundus. A bright-scan (B-scan) ultrasound revealed mild vitreous opacities with a vitreal membrane, shallow choroidal detachment, and no significant retinal and choroid layer thickening. The patient was admitted into the anterior segment division for anterior chamber (AC) tap culture and AC washout for suspected retained cortical matter removal, intracameral antibiotics (vancomycin and ceftazidime), and IOL explantation in the left eye. The initial aqueous tap culture had no growth. Nine days later, repeat aqueous tap and pupillary membrane cultures were positive for *Aspergillus spp. *Intravitreal voriconazole was administered along with topical natamycin and amphotericin B eye drops. There was a resolution of the clinical picture, and three weeks later a rebound occurred, for which AC washout, pars plana vitrectomy (PPV), capsulectomy, and intravitreal vancomycin, ceftazidime, and voriconazole were given. A week later, the patient developed a fungal granuloma behind the iris, which was successfully managed with an AC washout, removal of the granuloma, and repeated intravitreal and intracameral voriconazole administration. The best-corrected visual acuity (BCVA) during the last visit was 20/80 in the affected eye, with a plan for a secondary IOL implant.

Endophthalmitis is a rare but serious intraocular infection, with fungal endophthalmitis having a lower prevalence than bacterial endophthalmitis, which explains the lack of well-established guidelines for diagnosing and managing exogenous fungal endophthalmitis. This case highlights the rare presentation of post-cataract *Aspergillus *endophthalmitis with a fungal iris granuloma and demonstrates how the chronicity of this infection, along with surgical manipulation, may accelerate the seeding of these organisms into the anterior chamber structures.

## Introduction

Endophthalmitis is a devastating intraocular infection caused by bacteria or fungi. The prevalence of fungal endophthalmitis is generally lower than bacterial endophthalmitis [1]. The incidence of post-cataract endophthalmitis is between 0.03% and 0.2% worldwide [2]. However, the incidence rate of post-cataract fungal endophthalmitis is lower, ranging between 0.002% and 0.005% in developed countries [3], explaining the lack of treatment guidelines in the literature. Clinical signs of fungal endophthalmitis include eyelid edema, conjunctival congestion, anterior chamber cellular inflammation with or without hypopyon, and vitreal exudation [1]. Moreover, iris abscesses are rare [4], making diagnosis, as in this case, challenging. To our knowledge, a few cases of endophthalmitis presenting with iris abscesses have been reported in the literature, with only one related to *Aspergillus *[4,5].

The visual prognosis is generally poor, especially with exogenous causes of endophthalmitis [1]. Of the limited studies available, most covered endogenous fungal endophthalmitis. We share our experience in managing post-cataract *Aspergillus *endophthalmitis with fungal granuloma behind the iris.

## Case presentation

A 67-year-old female with diabetes mellitus with no predisposing factors like organ transplant, renal failure, dialysis, or other immunosuppressive therapy presented to the emergency department of King Khaled Eye Specialist Hospital, Riyadh, with a history of uncomplicated cataract surgery and intraocular lens implantation in the left eye approximately one month prior to symptom onset. Three days before presenting to our emergency department, the patient visited an emergency department at another center and was started on topical fortified ceftazidime and cefazoline eye drops every one hour, prednisolone acetate eye drops every two hours, atropine eyedrops three times a day (TID), and oral ciprofloxacin 500 mg twice daily (BID). Due to a lack of response, the patient was referred to our center for further management as a case of suspected retained lens material.

**Figure 1:**
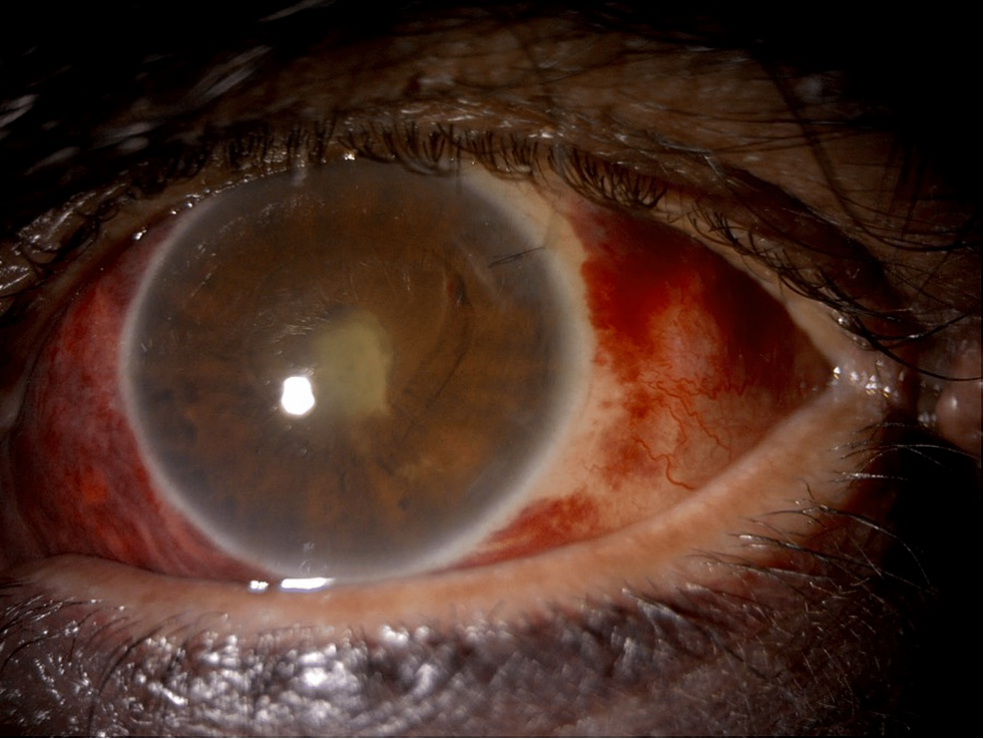
Slit lamp photo showing subconjunctival hemorrhage, mild corneal edema, central fibrinous reaction, and pupillary membrane formation.

On examination, visual acuity without correction was 20/25 in the right eye and hand motion in the left eye. The intraocular pressure measured by Tono-Pen (Reichert, Depew, NY, USA) was 14 mmHg and 8 mmHg in the right and left eyes, respectively. External examination revealed left lower eyelid ecchymosis, mild eyelid edema, and no tenderness. Slit-lamp and fundus examinations of the right eye revealed a normal anterior segment. In contrast, the examinations of the left eye revealed subconjunctival hemorrhage of approximately 360 degrees, a buried 10-0 nylon suture with a negative Seidel test, mild corneal edema with Descemet’s folds, a deep anterior chamber, no cells or hypopyon, a central fibrinous reaction causing pupillary membrane formation, posterior synechiae from six to nine o'clock, and an in situ intraocular lens (IOL) with no view to the fundus (Figure 1).

**Figure 2:**
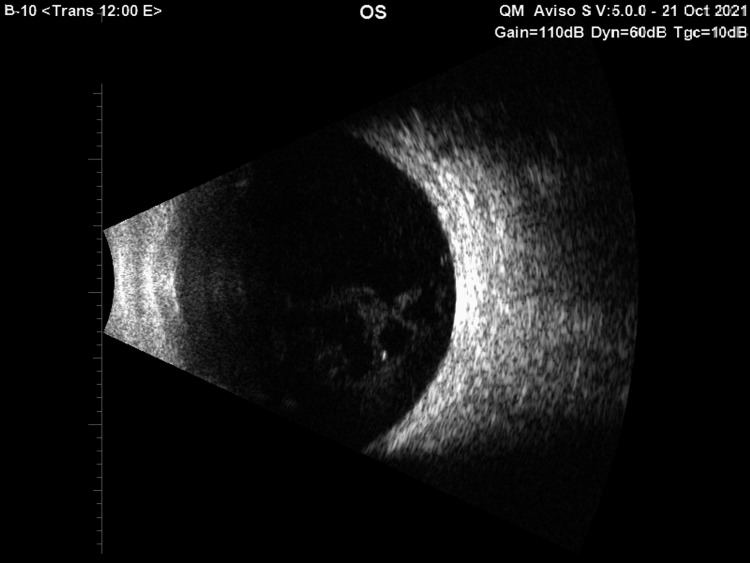
A bright scan (B-scan) ultrasound image showing mild vitreous opacities with cavitations and no significant retinal or choroidal thickening

The bright scan(B-scan) ultrasound showed mild vitreous opacities with cavitations, shallow choroidal detachment, and no significant retinal or choroid layer thickening (Figure 2).

**Figure 3:**
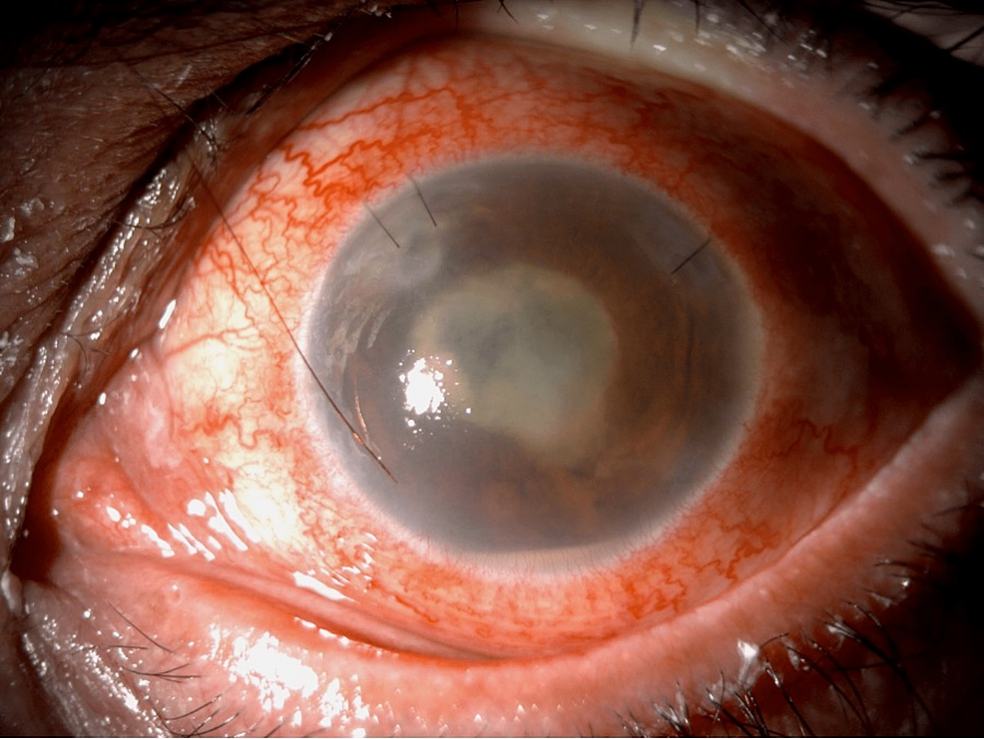
Slit lamp photo showing aphakia, hypopyon, fibrinous reaction, a pupillary membrane, and corneal infiltrate at the superonasal wound seven days after anterior chamber (AC) washout and intraocular lens (IOL) removal.

The patient was admitted into the anterior segment division for anterior chamber (AC) tap culture and AC washout for suspected retained cortical matter removal, intracameral antibiotics (vancomycin 1 mg/0.1 mL and ceftazidime 2.25 mg/0.1 mL), and IOL explantation in the left eye. Intraoperative findings revealed a dense fibrinous membrane in the anterior chamber, covering the entirety of the IOL with posterior synechiae. No retained lens material was found. The patient was switched to topical moxifloxacin eye drops four times a day (QID), oral moxifloxacin 400 mg once daily, topical prednisolone acetate eye drops QID, and topical atropine eye drops TID. Postoperative examination of the left eye revealed no eyelid swelling or tenderness, subconjunctival hemorrhage, moderate corneal edema, increased fibrinous reaction with a reformation of the pupillary membrane, localized inferotemporal peripheral anterior synechia, or an intraocular pressure of 20 mmHg. A B-scan revealed no changes compared to the previous examination. The aqueous culture showed no growth. On day seven post-AC washout, the patient developed a hypopyon with an increasing fibrinous reaction and a pupillary membrane (Figure 3).

**Figure 4:**
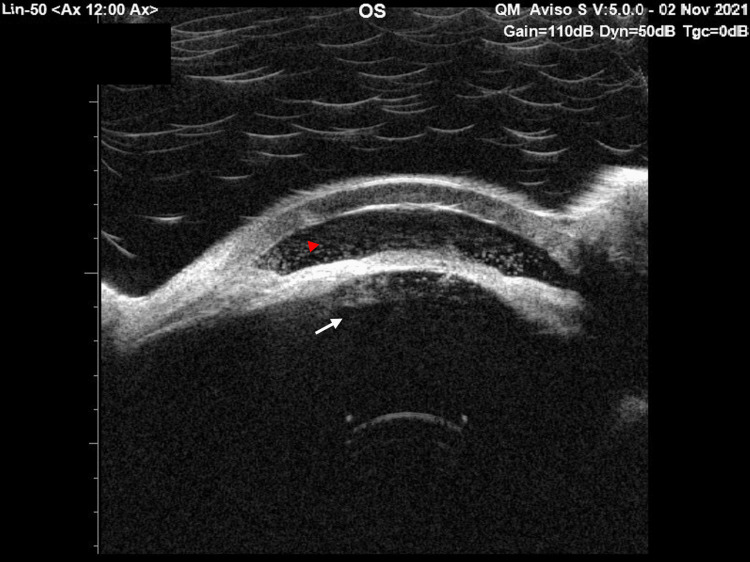
Ultrasound biomicroscopy image showing a shallow anterior chamber with diffuse opacities (red arrowhead) and multiple membranes behind the lens capsule (white arrow).

The B-scan follow-up revealed no changes. Ultrasound biomicroscopy (UBM) of the left eye indicated shallow AC with diffuse opacities and multiple membranes behind the lens capsule (Figure 4). 

**Figure 5:**
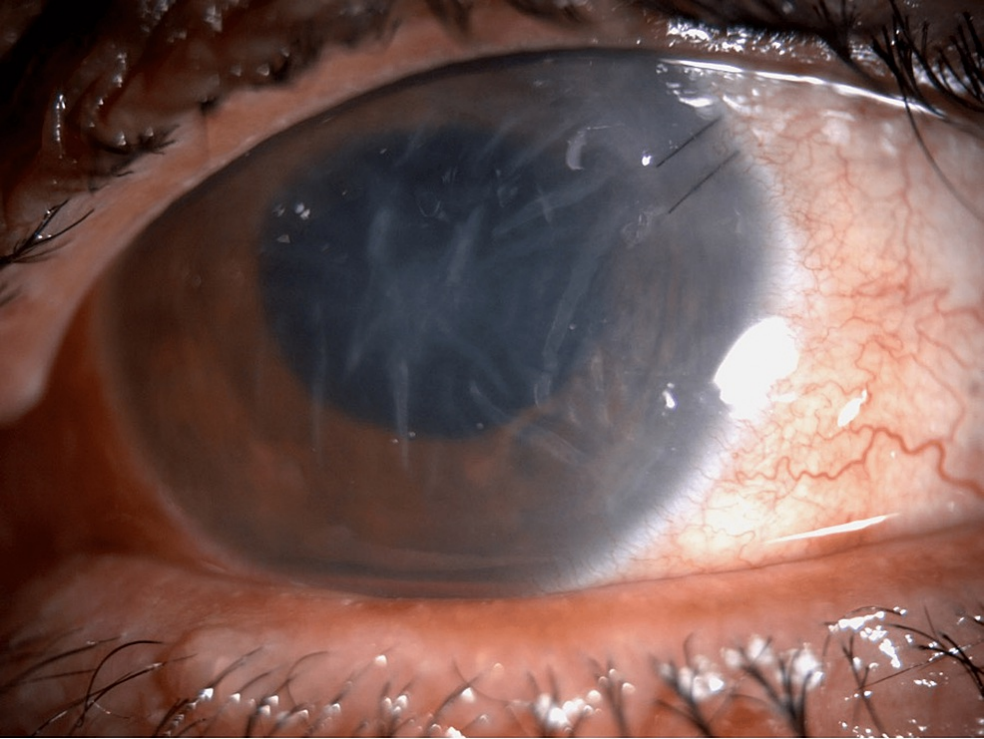
Slit lamp photo showing resolution of hypopyon and post-intravitreal voriconazole injection

Topical tissue plasminogen activator eye drops were statically administered. On day nine, post-AC washout, the patient was returned to the operating room for left-eye AC washout, membranectomy, anterior vitrectomy, and intracameral antibiotic (vancomycin 1 mg/0.1 mL and ceftazidime 2.25 mg/0.1 mL) injection. The cultures of the aqueous sample and pupillary membrane grew *Aspergillus spp*. The patient was started on topical amphotericin B (10 mg/mL drops), natamycin (5%) eye drops every hour, and oral voriconazole 200 mg BID and was referred to a vitreoretinal surgery team for culture-positive fungal endophthalmitis. Intravitreal voriconazole (100 mg/0.1 mL) was administered to the patient’s left eye. By day three post-intravitreal voriconazole, the hypopyon had resolved and the fundus view had improved (Figure 5).

The patient was discharged the next day and provided topical amphotericin B and natamycin eye drops every four hours, topical moxifloxacin eye drops every four hours, and oral voriconazole 200 mg BID.

**Figure 6:**
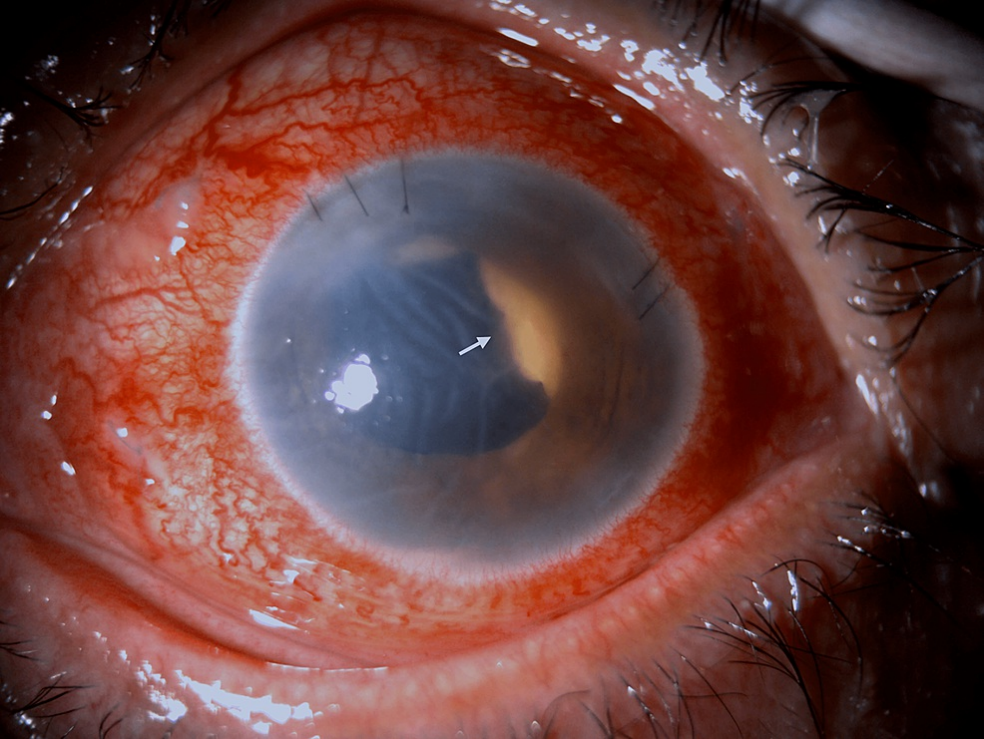
Slit lamp photo showing aphakia, a fungal granuloma behind the iris (white arrow).

**Figure 7:**
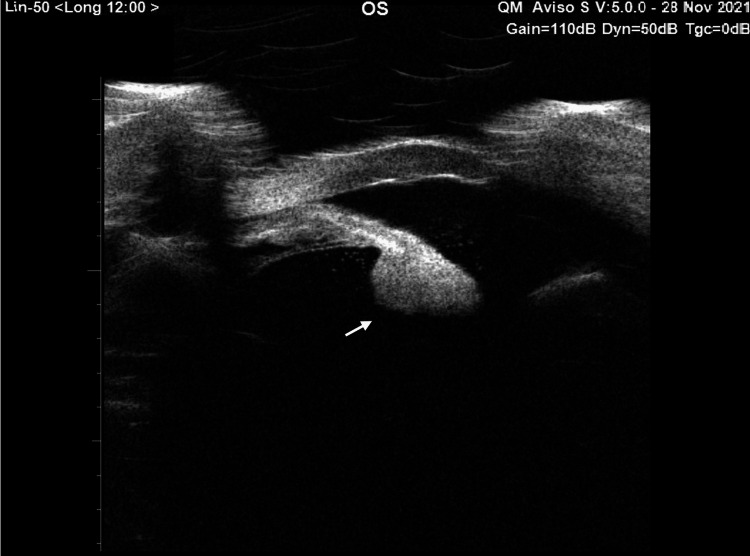
Ultrasound biomicroscopy image showing granuloma formation behind the iris (white arrow).

Ten days later, the patient was readmitted with recurrent pain in the left eye. Examination revealed mild corneal edema and a hypopyon 2 mm in height. Fundus examination revealed inferior vitreous seeding. The patient underwent left-eye AC washout, lens capsule removal, and pars plana vitrectomy (PPV). Intravitreal voriconazole, vancomycin, and ceftazidime injections were administered. On postoperative day five, a recurrence of the anterior segment inflammation with fungal granuloma formation behind the iris was observed and confirmed by UBM (Figures 6-7).

The patient underwent a left-eye AC washout with careful granuloma removal with a Simcoe cannula and intracameral voriconazole and amphotericin B injections. The patient was discharged two days later on topical voriconazole 1% drops every four hours and moxifloxacin 0.5% drops every six hours, with a one-week follow-up in the outpatient department.

**Figure 8:**
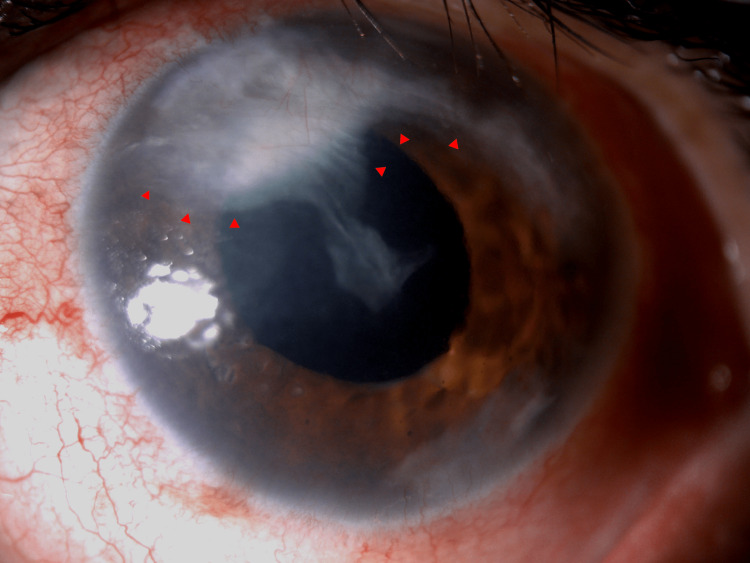
Slit lamp photo showing aphakia, corneal neovascularization, and fibrovascular membrane along the endothelium extending from 11-2 o’clock (red arrowheads).

During her last visit to the outpatient department, one year after iris granuloma removal, her best corrected visual acuity in the right eye was 20/30, in the left eye was 20/80, and the intraocular pressure was 15 mmHg in the right eye and 17 mmHg in the left eye. A slit-lamp examination revealed a normal anterior segment in the right eye, and the left eye revealed corneal neovascularization, scattered pigmented keratic precipitates, a fibrovascular membrane along the endothelium at 11 to two o’clock, slightly approaching the visual axis, aphakia, a quiet anterior chamber, and a normal fundus (Figure 8).

**Figure 9:**
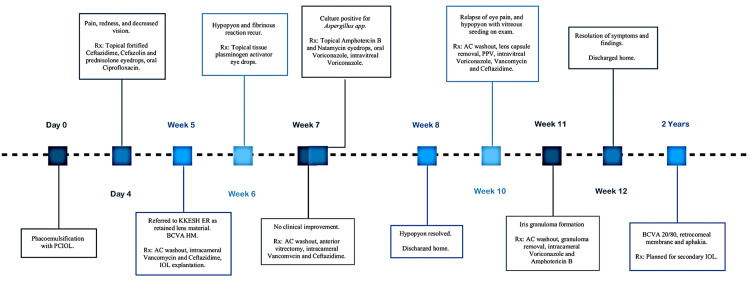
Timeline of interventions Rx: medical prescription; AC: anterior chamber; PPV: pars plana vitrectomy; PCIOL: posterior chamber intraocular lenses; KKESH ER: King Khaled Eye Specialist Hospital emergency room; BCVA: best-corrected visual acuity; HM: hand motion; IOL: intraocular lens

Corneal sutures were removed three months after discharge, and the patient was maintained on topical cyclosporine 1% drops every six hours, subconjunctival bevacizumab (2.5 mg/0.1 ml) was given once for corneal neovascularization regression with no effect. Currently, the patient is pending a secondary IOL implant. A timeline of the interventions for the patient's treatment is highlighted in Figure 9.

## Discussion

Endophthalmitis following cataract surgery is a rare, sight-threatening condition [6]. Fungal endophthalmitis is reportedly more common in tropical countries like India than in Western regions [7]. It has a prolonged latency period of weeks to months following intraocular inoculation, with an average latency period of seven weeks [8,9]. 

We describe the case of a 67-year-old patient with type 2 diabetes mellitus who developed non-specific intraocular inflammation one month after uneventful cataract surgery. Anterior chamber washout, IOL removal, anterior vitrectomy, and intravitreal antibiotics had optimal responses initially, but numerous relapses required a PPV with capsular bag removal to control the infection. The diagnosis of fungal endophthalmitis was not suspected initially due to the subacute presentation and lack of typical symptoms and signs, leading to a delay in the diagnosis.

The clinical presentation of exogenous fungal endophthalmitis varies, ranging from the classic triad of decreased vision, red eye, and ocular pain to insidious, non-specific ocular signs and progressive vision loss [9]. Intraocular inflammation is usually confined to one space - the anterior or posterior segment - whereas the inflammation is typically diffuse in bacterial endophthalmitis [9]. Examination findings vary from minimal anterior chamber inflammation to complete anterior chamber hypopyon [1]. Previous clinical and histopathologic reports highlight that infiltrates can localize to the anterior chamber, pupillary space, or anterior vitreous [8]. *Aspergillus*-mediated endophthalmitis is associated with more widespread retinal necrosis and retinal hemorrhages [1,4]. An iris abscess is rare [4,5], making this case diagnostically challenging.

Essentially, endophthalmitis is a clinical diagnosis with or without supportive testing. However, due to the subacute presentation in our case and responsiveness to early treatment measures, endophthalmitis was not initially suspected. The diagnostic yield of cultured vitreous fluid from a vitrectomy biopsy is suggested to be lower for fungal than bacterial endophthalmitis, with 20%-30% of cases having negative cultures [4, 9]. This explains why a definitive diagnosis was not reached with initial cultures in our case and supports the need for repeated cultures with a high clinical suspicion to get the final diagnosis. Molecular biology-based diagnostic methods, such as polymerase chain reaction, have high diagnostic potential; however, they are less practical in hospital settings and are prone to processing errors [1]. 

The source of infection is often the ocular surface in exogenous endophthalmitis [10]. Other sources include contaminated surgical equipment, air conditioning units, and hospital construction activities [6]. The patient’s surgery was performed in a facility in Jeddah, a city known for its humid climate and where a previous outbreak of ocular aspergillosis after cataract surgery had been described [11]. They concluded that this region's atmospheric temperature and humidity were ideal for the growth of *Aspergillus spp*. As for the most common isolate in postoperative fungal endophthalmitis, one study found filamentous fungi, mainly *Aspergillus* and *Fusarium *species, in 85% of cases and *Candida *species in the remaining 15% [1,9,12].

The main treatment for endophthalmitis involves intravitreal therapy with or without systemic antibiotics; however, surgical management is indicated in some cases. The Endophthalmitis Vitrectomy Study (EVS) reported that only patients who present with visual acuity of light perception (LP) or worse, benefit from immediate vitrectomy [13]. Regarding *Aspergillus *endophthalmitis, the Infectious Diseases Society of America (IDSA) guidelines advocate for systemic oral or intravenous voriconazole, intravitreal voriconazole, or intravitreal amphotericin B deoxycholate [1]. Amphotericin B is associated with higher resistance rates than voriconazole [1]. No consensus guidelines on the role of PPV exist for the treatment of fungal endophthalmitis. Theoretically, PPV decreases the infection load and increases the availability of antifungal agents in the retina [1]. In a retrospective case series, Dave et al. [14] advocate the benefits of early vitrectomy and intravitreal voriconazole, as it is reportedly less toxic to the retina.

Intraocular lens explantation may play a role in treating endophthalmitis for bacterial and fungal etiologies, with mixed success [15]. In the literature, evidence suggests that fungal filaments survive on the IOL surface [16] and in the capsular bag [17], with one series reporting successful infection control with complete capsulectomy [7]. Durand et al. documented the persistence of infection despite IOL removal, several vitrectomies, and intravitreal amphotericin B injections. *Aspergillus *endophthalmitis required oral voriconazole with intravenous caspofungin to eliminate the fungus [18]. In this case, IOL explantation, limited anterior vitrectomy, and multiple intravitreal and intracameral antimicrobials were insufficient to control the infection. Perhaps multiple procedures can lead to trauma to the anterior segment structures, potentially allowing the seeding of fungal elements into these structures, resulting in recurrent episodes of challenging management. Thus, the infection will keep recurring until the primary source is eradicated. Ultimately, lens capsulectomy, PPV, and careful removal of the granuloma alone without iris excision led to the resolution of infection in this patient.

The visual prognosis is generally poor, especially with exogenous causes of endophthalmitis [1]. Corneal involvement has been associated with worse visual outcomes [7]. Although the cornea was not involved initially in this case, a retrocorneal fibrovascular membrane developed later. However, the patient maintained a reasonable visual outcome, with best-corrected visual acuity (BCVA) of 20/80 during the last follow-up.

## Conclusions

This case was a rare presentation of post-cataract *Aspergillus *endophthalmitis with fungal granulomas. Early recognition and treatment of fungal endophthalmitis are essential for preventing further complications. Chronic and repeated anterior chamber manipulation can lead to the seeding of fungal elements into its structures. Pars plana vitrectomy, intravitreal antifungals with IOL removal, and capsulectomy should be considered as initial treatments in suspicion of post-cataract fungal endophthalmitis. More studies with a larger sample size to explore the role of early PPV in exogenous fungal endophthalmitis are needed.
